# High‐density lipoprotein protects cardiomyocytes from oxidative stress via the PI3K/mTOR signaling pathway

**DOI:** 10.1002/2211-5463.12279

**Published:** 2017-08-14

**Authors:** Manabu Nagao, Ryuji Toh, Yasuhiro Irino, Hideto Nakajima, Toshihiko Oshita, Shigeyasu Tsuda, Tetsuya Hara, Masakazu Shinohara, Tatsuro Ishida, Ken‐ichi Hirata

**Affiliations:** ^1^ Division of Cardiovascular Medicine Kobe University Graduate School of Medicine Japan; ^2^ Division of Evidence‐Based Laboratory Medicine Kobe University Graduate School of Medicine Japan; ^3^ Division of Epidemiology Kobe University Graduate School of Medicine Japan

**Keywords:** high‐density lipoprotein, mTOR signaling, oxidative stress

## Abstract

Low levels of plasma high‐density lipoprotein (HDL) cholesterol are associated with an increased risk of heart failure, regardless of the presence or absence of coronary artery disease. However, the direct effects of HDL on failing myocardium have not been fully elucidated. We found that HDL treatment resulted in improved cell viability in H9c2 cardiomyocytes under oxidative stress. This cardioprotective effect of HDL was regulated via the phosphatidylinositol 3‐kinase (PI3K)/mammalian target of rapamycin (mTOR) pathway. mTOR signaling promotes cell survival through the inactivation of the BCL2‐associated agonist of cell death via phosphorylation of ribosomal protein S6 kinase. Modulation of cardiac PI3K/mTOR signaling by HDL could represent a novel therapeutic strategy for heart failure.

**Abbreviations**
HDL: high‐density lipoprotein
mTOR: mammalian target of rapamycin
PI3K: phosphatidylinositol 3‐kinase

Heart failure is a worldwide public health problem, and its prevention and treatment should be of high priority to our society 1, 2. According to the American Heart Association, there are around 550 000 new patients with heart failure each year 1. The inverse relationship between circulating high‐density lipoprotein (HDL) cholesterol level and incidence of cardiovascular disease has been demonstrated in a number of clinical trials 3, 4, 5, 6. It has been also demonstrated that lower plasma HDL levels are associated with increased risk of heart failure 7, 8, 9. Efflux of cholesterol from macrophages and its return to the liver, known as reverse cholesterol transport, is one of the most important mechanisms by which HDL suppresses the progression of atherosclerosis 10, 11. Additionally, HDL is well known to have anti‐inflammatory, antioxidative, and antiapoptotic effects in the cardiovascular system 12. Interestingly, Frias *et al*. and others have reported that sphingosine‐1‐phosphate (S1P), a component of HDL particles, protects cardiomyocytes against myocardial ischemia/reperfusion injury 13, 14, 15. However, beyond its anti‐ischemic and antiatherogenic properties, the direct effects of HDL on heart failure have not been fully elucidated.

The serine threonine protein kinase mammalian target of rapamycin (mTOR) is a critical regulator of protein synthesis and controls cell survival, growth, and proliferation 16. mTOR belongs to phosphatidylinositol 3‐kinase (PI3K)‐related kinase family and interacts with a large number of proteins to form two different complexes named mTOR complex 1 (mTORC1) and 2 (mTORC2) 17. These complexes modulate many major cellular functions and are associated with several pathological conditions, including cancer, obesity, type 2 diabetes, and neurodegeneration 17, 18. In this study, we therefore sought to determine whether HDL exerts cardioprotective effects on cardiomyocytes under oxidative stress via the mTOR signaling pathway.

## Materials and methods

### Cell culture

Rat cardiomyoblast cells (H9c2) were purchased from the European Collection of Authenticated Cell Cultures (ECACC) and maintained in Dulbecco's modified Eagle's medium (DMEM; Wako, Tokyo, Japan) supplemented with 10% fetal bovine serum and 100 U·mL^−1^ penicillin/streptomycin (both from Sigma‐Aldrich, St. Louis, MO, USA) at 37 °C and 5% CO_2_. For all experiments, cells were serum‐starved overnight. LY294002 (Cell Signaling Technology, Danvers, MA, USA) and rapamycin (Wako) were added 1 h before the treatment of HDL.

### HDL preparation

Human HDL used in this study was purchased from EMD Millipore (Temecula, CA, USA). According to the manufacturer's information, HDL was isolated using KBr density gradient ultracentrifugation technique, and the purity of more than 95% was confirmed by SDS/PAGE.

### Western blotting

Western blot analysis was performed as previously reported 19. Briefly, cells were washed with cold phosphate‐buffered saline, and the proteins were extracted with ice‐cold lysis buffer (20 mm 4‐(2‐hydroxyethyl)‐1‐piperazineethanesulfonic acid, pH 7.4, 150 mm NaCl, 1% sodium dodecyl sulfate, and 1% nonyl phenoxypolyethoxylethanol (NP‐40)) and separated by 8–15% sodium dodecyl sulfate/polyacrylamide gel electrophoresis. Gels were transferred to polyvinylidene difluoride membranes (Immobilon‐P; Millipore, Billerica, MA, USA) and blocked in 5% (w/v) milk in Tris‐buffered saline with Tween 20. After blocking, the membranes were incubated with primary antibodies overnight at 4 °C and then with horseradish peroxidase‐conjugated secondary antibodies for 1 h at room temperature. The blots were enhanced with either Luminata™ Forte Western HRP Substrate (Millipore, USA) or SuperSignal West Pico chemiluminescence substrate (Thermo Scientific, Waltham, MA, USA), and detected using an Amersham Imager 600 (GE Healthcare, Tokyo, Japan). The antibodies used in this study were as follows: phospho‐Akt (Ser473), Akt, phospho‐p70 S6K (Thr389), p70 S6K (Cell Signaling Technology), phospho‐BAD (Ser136) (GeneTex, Irvine, CA, USA), BAD (Abcam, Cambridge, MA, USA), and β‐actin (Sigma‐Aldrich). Band quantification was performed using imagej ^®^ software (National Institutes of Health, USA).

### Cell viability assay

H9c2 cells were cultured into 96‐well plates at 5 × 10^3^ cells per well. After serum starvation and pretreatment with 50, 100, or 200 μg·mL^−1^ HDL overnight with or without 50 μm LY294002 or 10 nm rapamycin, the cells were stimulated with 100 μm H_2_O_2_ for 2 h. Cell viability was measured using a commercially available assay (Cell Counting Kit‐8; Dojindo, Kumamoto, Japan).

### Fluorescent imaging of cell death

H9c2 cells were seeded on eight‐well chamber slides (Nunc^®^ Lab‐TEK™ Chamber Slide™; Sigma‐Aldrich) at 2 × 10^4^ cells per well. Cells were serum‐starved and treated with 100 μg·mL^−1^ HDL overnight with or without 10 nm rapamycin, and then stimulated with 100 μm H_2_O_2_ for 2 h. Dead cells were detected using propidium iodide (Takara, Kusatsu, Japan) according to the manufacturer's protocol. The images were acquired with a fluorescence microscope (BZ‐X700 microscope; Keyence, Osaka, Japan).

### Caspase 3 activity assay

H9c2 cells were seeded into 3 × 10^6^ cells/150‐mm culture dishes and incubated in DMEM containing 10% fetal bovine serum at 37 °C and 5% CO_2_. After 24 h of incubation, cells were subjected to serum starvation and treated with 100 μg·mL^−1^ HDL overnight with or without 10 nm rapamycin. After 2 h of stimulation with 100 μm H_2_O_2_, cells were harvested, and caspase 3 activity was measured using a commercially available assay (Caspase 3 Assay kit; Abcam) according to the manufacturer's protocol.

### RNA interference

SR‐BI‐targeting and negative control small interfering RNA (siRNA) were purchased from SIGMA Genosys (#1; Rn_Scarb1_9396_s and Rn_Scarb1_9396_as, #2; Rn_Scarb1_9397_s and Rn_Scarb1_9397_as, negative control; SIC‐001_s and SIC‐001_as) and Invitrogen (Yokohama, Japan) respectively. The siRNA were transfected into H9c2 cells using Lipofectamine RNAiMAX reagent (Thermo Fisher Scientific, Yokohama, Japan) according to the manufacturer's protocol. After 48 h of transfection, the medium was changed to the experimental medium.

### Statistical analysis

Data among multiple groups were tested by one‐way ANOVA followed by Tukey's multiple comparison test. Differences in means between two groups were evaluated using an unpaired two‐tailed Student's *t*‐test. Statistical significance was determined as *P* values < 0.05 (*P < 0.05; **P < 0.01). All statistical analyses were conducted using graphpad prism software (La Jolla, CA, USA).

## Results

### HDL protects cardiomyocytes from oxidative stress

**Figure 1:**
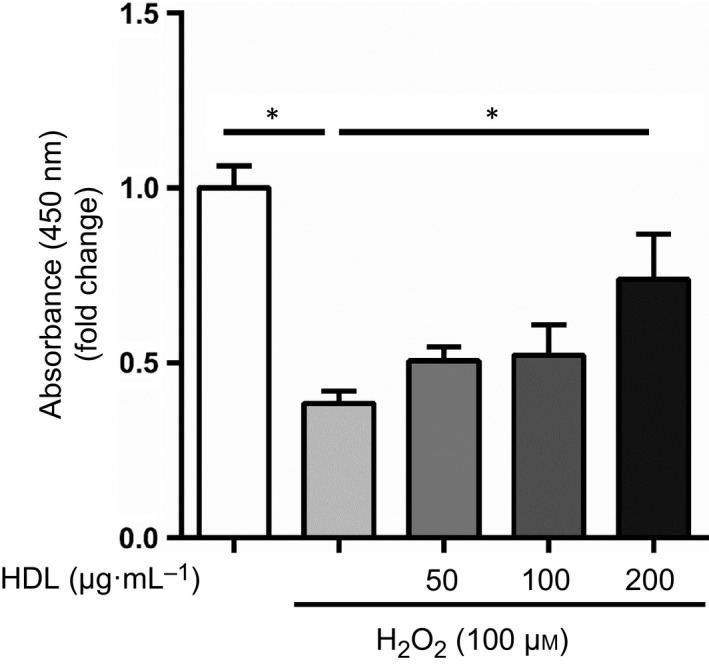
HDL protects cardiomyocytes from oxidative stress. H9c2 cells were serum‐starved and incubated with 50, 100, or 200 μg·mL^−1^ HDL overnight and then stimulated with 100 μm H_2_O_2_ for 2 h. Cell viability was assessed using a cell counting kit (*n* = 5 in each group). *, *P* < 0.05 by one‐way ANOVA followed by Tukey's multiple comparison test. All values are mean ± SEM.

There is a strong, well‐established relationship between the levels of myocardial reactive oxygen species 18, cardiomyocyte damage due to oxidative stress, and left ventricular contractile dysfunction, leading to heart failure 20, 21. To assess the effects of HDL against oxidative stress in cardiomyocytes, we incubated H9c2 cells with H_2_O_2_ after pretreatment with HDL, and analyzed cell viability. HDL protected cells from oxidative stress in a dose‐dependent manner (Fig. 1).

### HDL cardioprotective effects are mediated by the PI3K/mTOR signaling pathway

**Figure 2:**
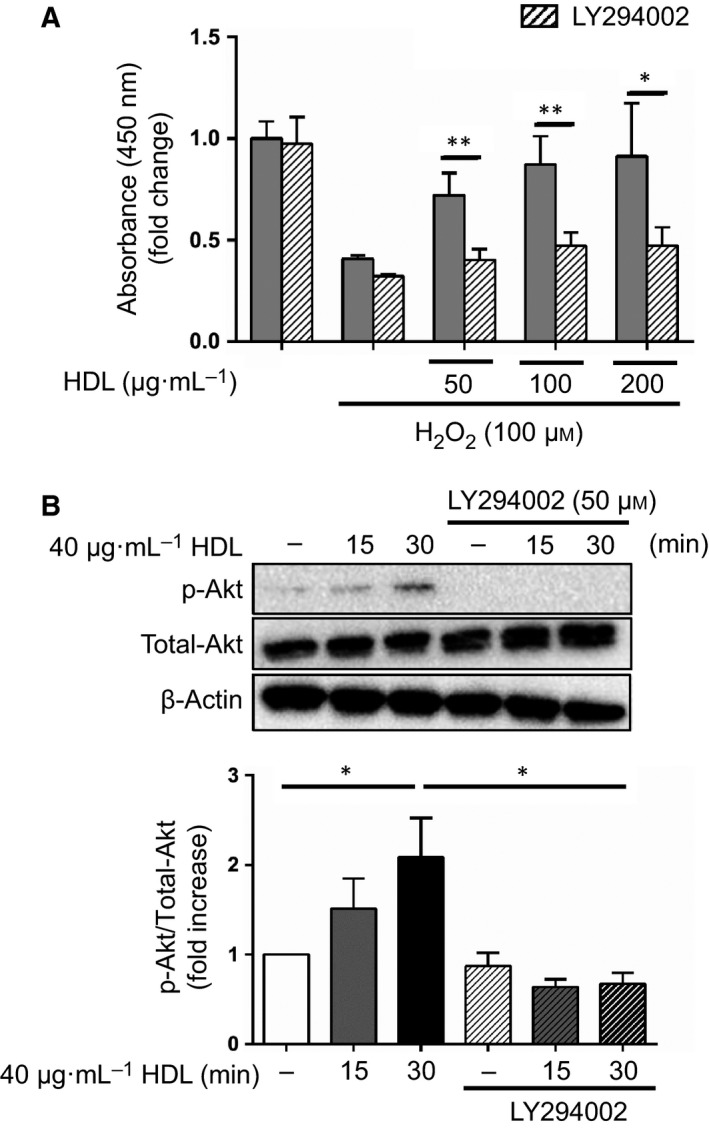
The cardioprotective effects of HDL are mediated by the PI3K signaling pathway. (A) After serum starvation and incubation with 50, 100, or 200 μg·mL^−1^ HDL overnight, H9c2 cells were stimulated with 100 μm H_2_O_2_ for 2 h. The PI3K inhibitor LY294002 was added 1 h before incubation with HDL. Cell viability was assessed using a cell counting kit (*n* = 5 in each group). *, *P* < 0.05, **, *P* < 0.01 by unpaired two‐tailed Student's *t*‐test. All values are mean ± SEM. (B) Western blot analysis of total and phospho‐Akt in H9c2 cells. After overnight serum starvation, cells were treated with 40 μg·mL^−1^ HDL for 15 or 30 min with or without 50 μm LY294002, which was added 1 h before incubation with HDL. The band intensity of total and phospho‐Akt was quantified and normalized to β‐actin. Note that the higher exposure for the P‐Akt blot and the lower exposure for the total‐Akt and actin blots are presented for optimal contrast (*n* = 5 in each group). *, *P* < 0.05 by one‐way ANOVA followed by Tukey's multiple comparison test. All values are mean ± SEM.

**Figure 3:**
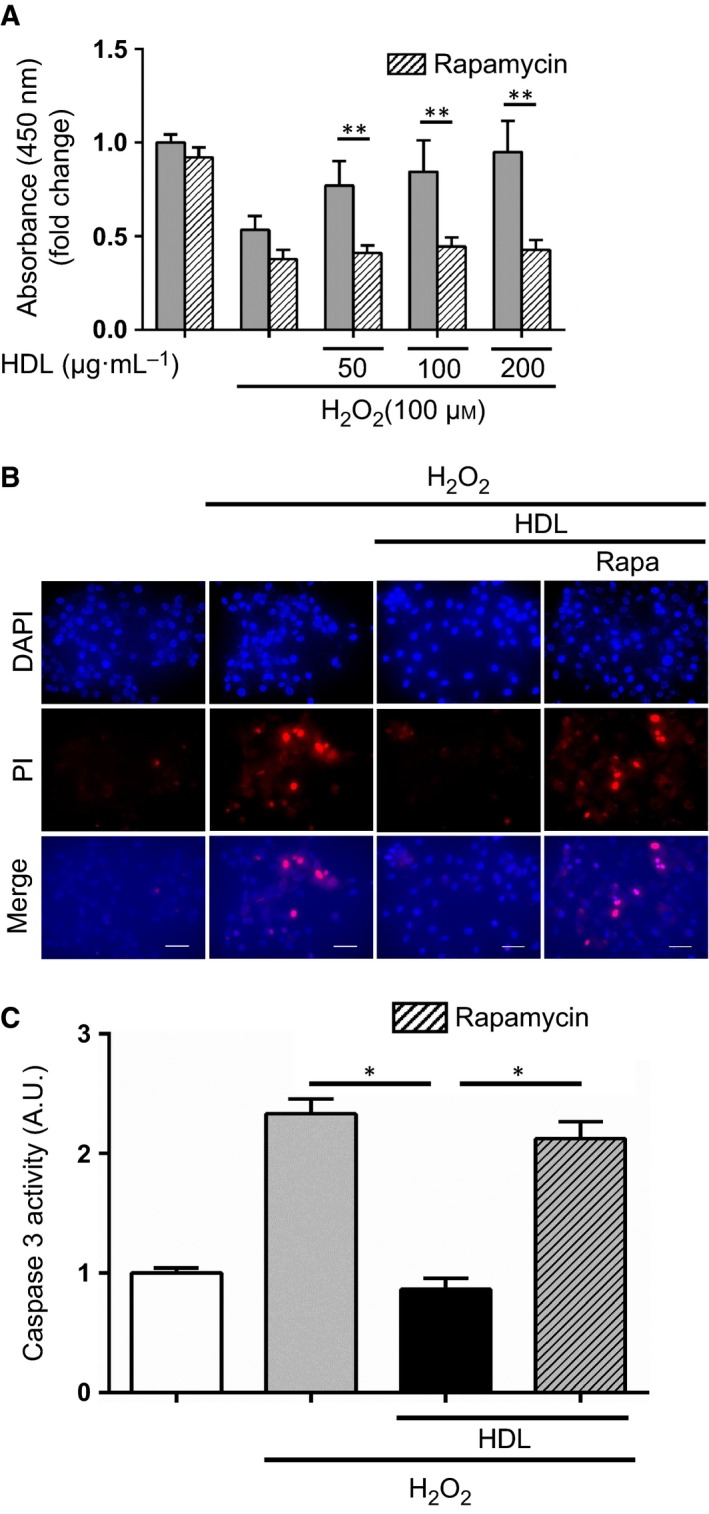
The cell survival effect of HDL depends on mTOR signaling. (A) After serum starvation and incubation with 50, 100, or 200 μg·mL^−1^ HDL overnight, H9c2 cells were stimulated with 100 μm H_2_O_2_ for 2 h. To inhibit mTOR, 10 nm rapamycin was added before incubation with HDL. Cell viability was assessed using a cell counting kit (*n* = 5 in each group). **, *P* < 0.01 by unpaired two‐tailed Student's *t*‐test. All values are mean ± SEM. (B) Propidium iodide staining of H9c2 cells. Cells were cultured in serum‐free medium with or without 10 nm rapamycin, treated with 100 μg·mL^−1^ HDL overnight, and then stimulated with 100 μm H_2_O_2_ for 2 h. The photographs were acquired with a fluorescence microscope. Images are representative of three independent experiments. Scale bars = 50 μm. (C) Caspase 3 activity of H9c2 cells under oxidative stress. Cells were seeded into 3 × 10^6^ cells/150‐mm culture dishes. After serum starvation, cells were treated with 100 μg·mL^−1^ HDL overnight and stimulated with 100 μm H_2_O_2_ for 2 h. To inhibit mTOR, 10 nm rapamycin was added 1 h before incubation with HDL. Caspase 3 activity was analyzed using caspase 3 assay kit (*n* = 3 in each group). *, *P* < 0.05 by one‐way ANOVA followed by Tukey's multiple comparison test. All values are mean ± SEM.

Phosphatidylinositol 3‐kinase/AKT signaling has been demonstrated to protect cardiomyocytes from ischemia/reperfusion injury 22. Therefore, we considered the possibility that, under oxidative stress, HDL may exert positive effects on the myocardium through the PI3K/Akt pathway. Consistent with this hypothesis, addition of the PI3K inhibitor LY294002 suppressed the cell survival effects of HDL (Fig. 2A). We also examined whether HDL activated the PI3K/Akt pathway using western blot analysis. Treatment of HDL significantly increased the phosphorylation of Akt in H9c2 cells, whereas treatment with LY294002 blocked HDL‐induced phosphorylation of Akt (Fig. 2B). To determine which pathway downstream of PI3K/Akt HDL was activating, we tested the contribution of mTOR signaling to the observed cytoprotective effects. Treatment with rapamycin, a classical mTOR inhibitor, significantly attenuated the cell survival effects of HDL treatment (Fig. 3A). Propidium iodide staining and caspase 3 activity assay revealed that HDL treatment protected against H_2_O_2_‐induced apoptosis, and rapamycin attenuated the antiapoptotic effects of HDL (Fig. 3B,C). These findings indicate that the PI3K/mTOR signaling pathway plays an important role in the cardioprotective effects of HDL during oxidative stress.

### HDL promotes the phosphorylation of S6 kinase and the proapoptotic protein BAD via the PI3K/mTOR signaling pathway

**Figure 4:**
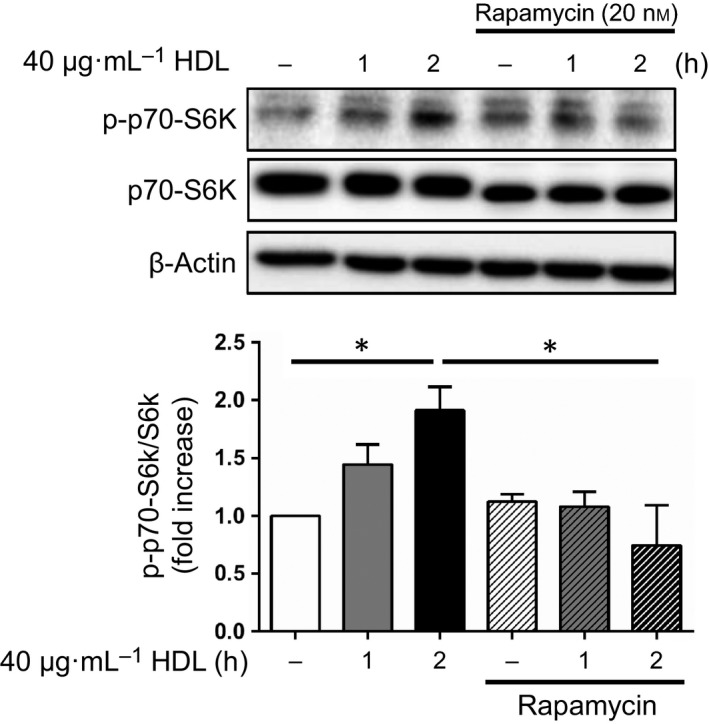
HDL upregulates the phosphorylation of S6K, the downstream kinase of mTOR. Western blot analysis of total and phospho‐S6K in H9c2 cells. After serum starvation, cells were incubated with 40 μg·mL^−1^ HDL for 1 or 2 h with or without 20 nm rapamycin. The images are representative of three independent experiments. The band intensity of phospho‐S6K was quantified using imagej software and normalized to S6K. *, *P* < 0.05 by one‐way ANOVA followed by Tukey's multiple comparison test. All values are mean ± SEM.

**Figure 5:**
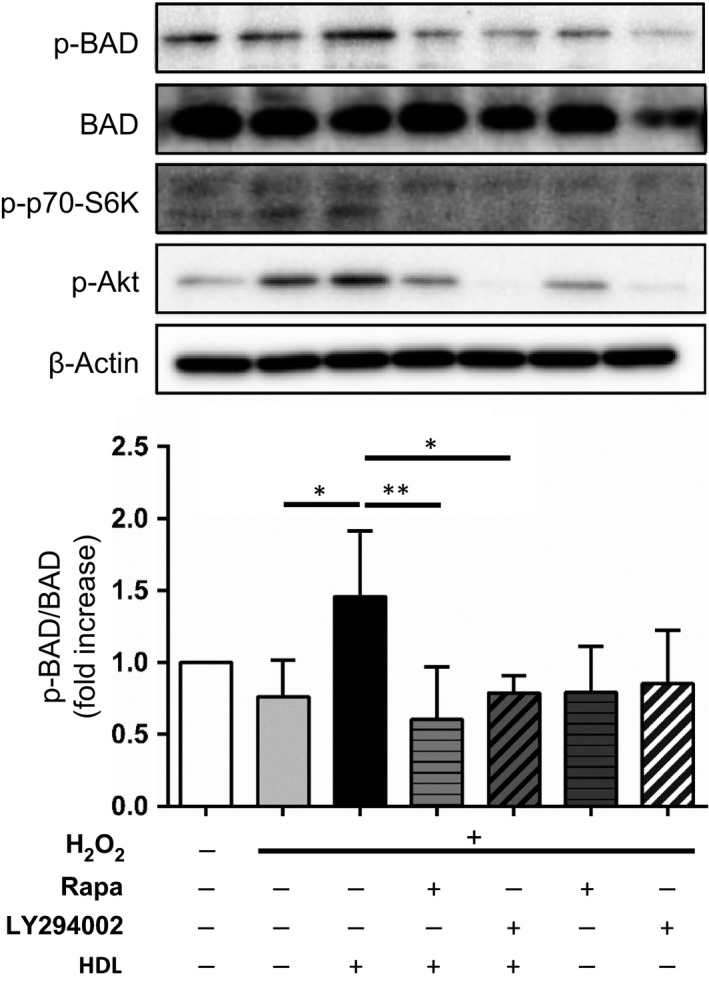
HDL treatment leads to the phosphorylation of S6K and BAD via the PI3K/mTOR signaling pathway. Western blot analysis of BAD, phospho‐BAD, phospho‐S6K, and phospho‐Akt in H9c2 cells. After serum starvation, cells were incubated with 40 μg·mL^−1^ HDL for 2 h with or without 50 μm LY294002 or 20 nm rapamycin, followed by 100 μm H_2_O_2_ stimulation for 1 h. The images are representative of four independent experiments. The band intensity of phospho‐BAD was quantified using imagej software and normalized to BAD. *, *P* < 0.05; **, *P* < 0.01 by one‐way ANOVA followed by Tukey's multiple comparison test. All values are mean ± SEM.

To assess the effects of HDL treatment downstream of mTOR signaling, we examined the phosphorylation state of the ribosomal protein S6 kinase (S6K), one of the most well‐characterized targets of mTORC1, by western blot analysis. HDL treatment led to increased phosphorylation of S6K, and this was suppressed by pretreatment with rapamycin (Fig. 4). mTORC1 signaling promotes cell survival through the inactivation of BCL2‐associated agonist of cell death (BAD), a proapoptotic BH3‐only member of the Bcl‐2 family, via phosphorylation by S6K 23, 24. As shown in Fig. 5, we also found that HDL treatment led to increased phosphorylation of BAD, as well as Akt and S6K, under H_2_O_2_ stimulation, and both LY294002 and rapamycin reduced HDL‐induced phosphorylation of BAD. As previously reported, the phosphorylation of Akt was upregulated to compensate for oxidative stress 25. Furthermore, HDL treatment strongly augmented the phosphorylation of Akt and S6K.

## Discussion

**Figure 6:**
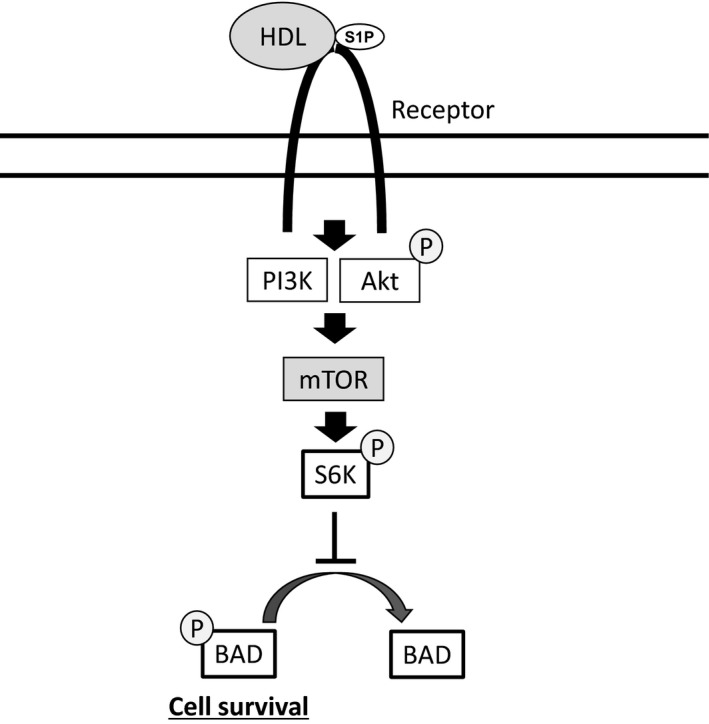
Proposed mechanism of HDL cardioprotection. HDL activates the PI3K/Akt signaling pathway, leading to the activation of mTOR. mTOR enhances the phosphorylation of S6K and BAD, which inhibits the proapoptotic function of BAD.

In this study, we have demonstrated that HDL treatment improves cardiomyocyte viability under oxidative stress and that the PI3K/mTOR signaling pathway mediates these effects. We have also revealed that S6K, a downstream effector kinase of mTOR signaling, is involved in antiapoptotic signaling through the maintenance of BAD phosphorylation, and HDL treatment, signaling through PI3K/mTOR, increased the phosphorylation of both S6K and BAD under oxidative stress (Fig. 6).

HDL is a complex particle containing a number of multifunctional proteins and lipids, and can activate intracellular signaling pathways in cardiomyocytes, leading to cardioprotection 26, 27. We sought to determine how HDL initially communicates with cardiomyocytes, and investigated the interaction with scavenger receptor type I (SR‐BI), a receptor involved in the uptake of cholesterol from HDL 28. SR‐BI is mainly expressed in hepatocytes, ovary, adrenal glands, and macrophages and also in cardiomyocytes 29. As the precise role of SR‐BI is not fully understood, particularly in cardiomyocytes, we conducted several experiments to investigate whether the interaction between SR‐BI and either HDL particles or apolipoprotein AI mediated cardioprotective effects. We could not find any evidence to support the involvement of SR‐BI in the cardioprotective effects of HDL (Figs S1–S3).

As mentioned above, S1P, a major constituent of HDL, binds to its receptors on the surface of myocardium, generating multiple downstream signals related to prosurvival proteins such as extracellular signal‐regulated kinase 1/2, the transcription factor signal transducer and activator of transcription 3, and PI3K/Akt 22, 30. For instance, in an *in vivo* transient ligating left coronary artery model, HDL injection reduced infarct size and necrotic cell death, and this was mediated via S1P‐dependent nitric oxide production 14, 31. S1P causes the activation of S1P(2) and S1P(3) receptors, leading to Akt phosphorylation. This S1P‐mediated Akt activation plays a significant role in protecting cardiomyocytes from ischemia/reperfusion damage *in vivo* 13. These studies indicate that the cardioprotection caused by HDL is partly due to S1P, and it has become a promising target in the treatment for ischemic heart disease. In the present study, S1P contained in HDL particles might act as the origin of the signal transduction pathway (Fig. 6).

Recently, several studies have identified mTOR as an important regulator of cardiac adaptation, as its overexpression was protective in pressure‐overloaded mouse hearts 18, 32, and a murine heart conditional knockout caused cardiac dysfunction 33. By generating a targeted myocardial deletion in raptor, a vital component of mTORC1, Shende *et al*. reported that mTORC1 activity was essential for the preservation of cardiac function. The deletion deteriorated cardiac function rapidly, resulting in dilated cardiomyopathy and high mortality 34. Similarly, they showed that mTORC2 was involved in maintaining left ventricular contractile function of pressure‐overloaded mouse hearts. Cardiac dysfunction caused by transverse aortic constriction was more severe after ablation of rictor, a specific component of mTORC2 35. Our work suggests that, in terms of cardiac protection, HDL could be an activator of mTOR signaling.

In the present study, we assessed the phosphorylation of S6K using the mTORC1 inhibitor rapamycin. It has been reported that long‐term incubation with rapamycin can also inhibit mTORC2 36, which directly activates Akt by phosphorylation of its hydrophobic motif, leading to cell survival 37. Hence, it is possible that the cardioprotective effects of HDL are due to not only mTORC1 but also mTORC2 and that both mTOR complexes contribute to cell survival in the heart.

In conclusion, the present study demonstrates that HDL protects cardiomyocytes from oxidative stress and that this effect is mediated through the PI3K/mTOR signaling pathway. This suggests that modulation of cardiac mTOR signaling by HDL could represent a novel therapeutic strategy for heart failure.

## Author contributions

MN, YI, and HN planned and performed the experiments and analyzed the data. MN and RT wrote and edited the manuscript. RT, TI, and KH designed the research and oversaw the progress of the experimental work. TO and ST performed the experiments. TH and MS contributed to the design and planning of experiments.

## Supporting information


**Fig. S1.** The cardioprotective effect of HDL was not affected by SR‐BI depletion.
**Fig. S2.** SR‐BI neutralizing antibody does not affect Akt phosphorylation.
**Fig. S3.** ApoAI does not affect cell survival in cardiomyocytes.
