# Supplementary material for: High‐density lipoprotein protects cardiomyocytes from oxidative stress via the PI3K/mTOR signaling pathway
Source: FEBS Open Bio. 2017 Aug 14;7(9):1402–9. doi: 10.1002/2211-5463.12279 (PMC5586351; doi:10.1002/2211-5463.12279)
Supplement: Supplementary file 1 — Fig. S1. The cardioprotective effect of HDL was not affected by SR‐BI depletion. Fig. S2. SR‐BI neutralizing antibody does not affect Akt phosphorylation. Fig. S3. ApoAI does not affect cell survival in cardiomyocytes. [file FEB4-7-1402-s001.pdf]

High Density Lipoprotein Protects Cardiomyocytes from Oxidative Stress via the PI3K/ mTOR Signaling Pathway

Manabu Nagao[1], Ryuji Toh[2], Yasuhiro Irino[2], Hideto Nakajima[1], Toshihiko Oshita[1], Shigeyasu Tsuda[1], Tetsuya Hara[1], Masakazu Shinohara[3], Tatsuro Ishida[1], and Ken-ichi Hirata[1, 2]

[1]Division of Cardiovascular Medicine, Kobe University Graduate School of Medicine
[2]Division of Evidence-Based Laboratory Medicine, Kobe University Graduate School of Medicine
[3]Division of Epidemiology, Kobe University Graduate School of Medicine

Supporting Information

Supplemental Figure 1-3.

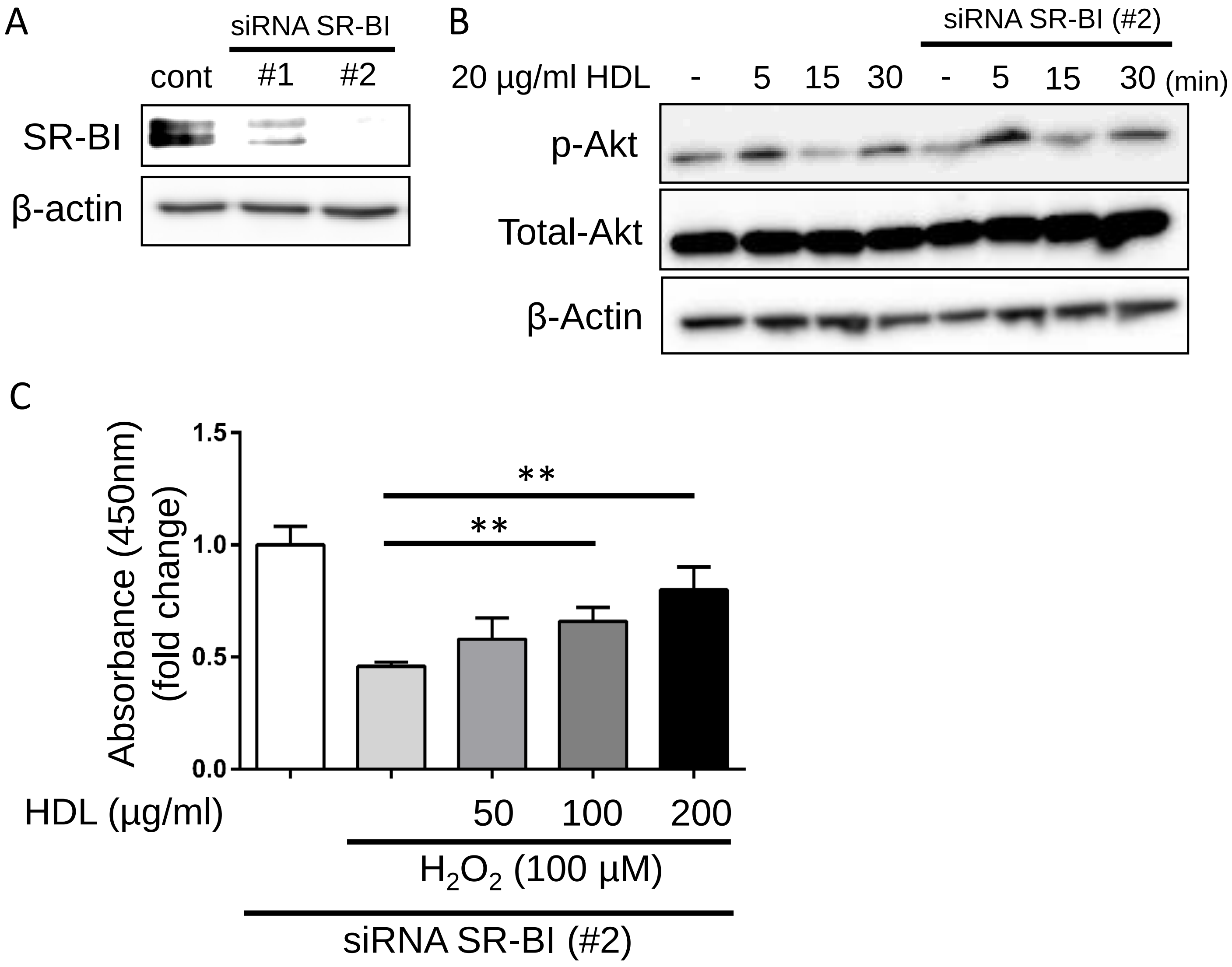

Supplemental Figure 1. **The cardioprotective effect of HDL was not affected by SR-BI depletion**

A, Western blot analysis of H9c2 cells following SR-BI knockdown. **B**, Western blot analysis of total and phospho-Akt in H9c2 cells. After siRNA-mediated SR-BI depletion, cells were treated with 20 μg/ml HDL for 5, 15, or 30 minutes with or without 50 μM LY294002. Data are representative of 2 independent experiments. **C**, H9c2 cells were transfected with either SR-BI or control siRNA, and 48 hours later, they were incubated with 50, 100, or 200 μg/ml HDL overnight, and then stimulated with 100 μM H_2O_2 for 2 hours. Cell viability was assessed using a cell counting kit. (n=5 in each group). *, $P < 0.05$ by one-way ANOVA followed by Tukey's multiple comparison test. All values are mean ± SD.

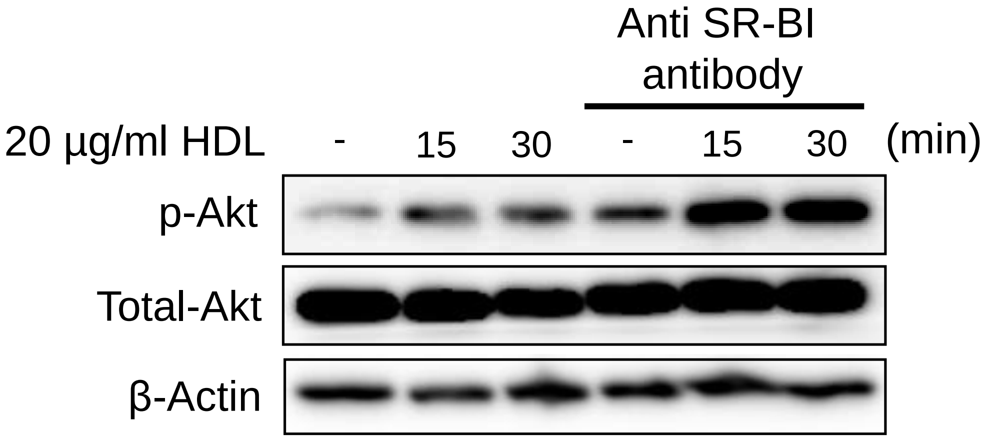

Supplemental Figure 2. **SR-BI neutralizing antibody does not affect Akt phosphorylation** Western blot analysis of total and phospho-Akt in H9c2 cells. β-Actin serves as a loading control. After serum starvation, cells were incubated with SR-BI neutralizing antibody (Novus Biologicals, USA) or normal rabbit IgG (Santa Cruz, USA) for 1 hour, followed by the addition of 20 μg/ml HDL for 15 or 30 minutes. Data are representative of 4 independent experiments.

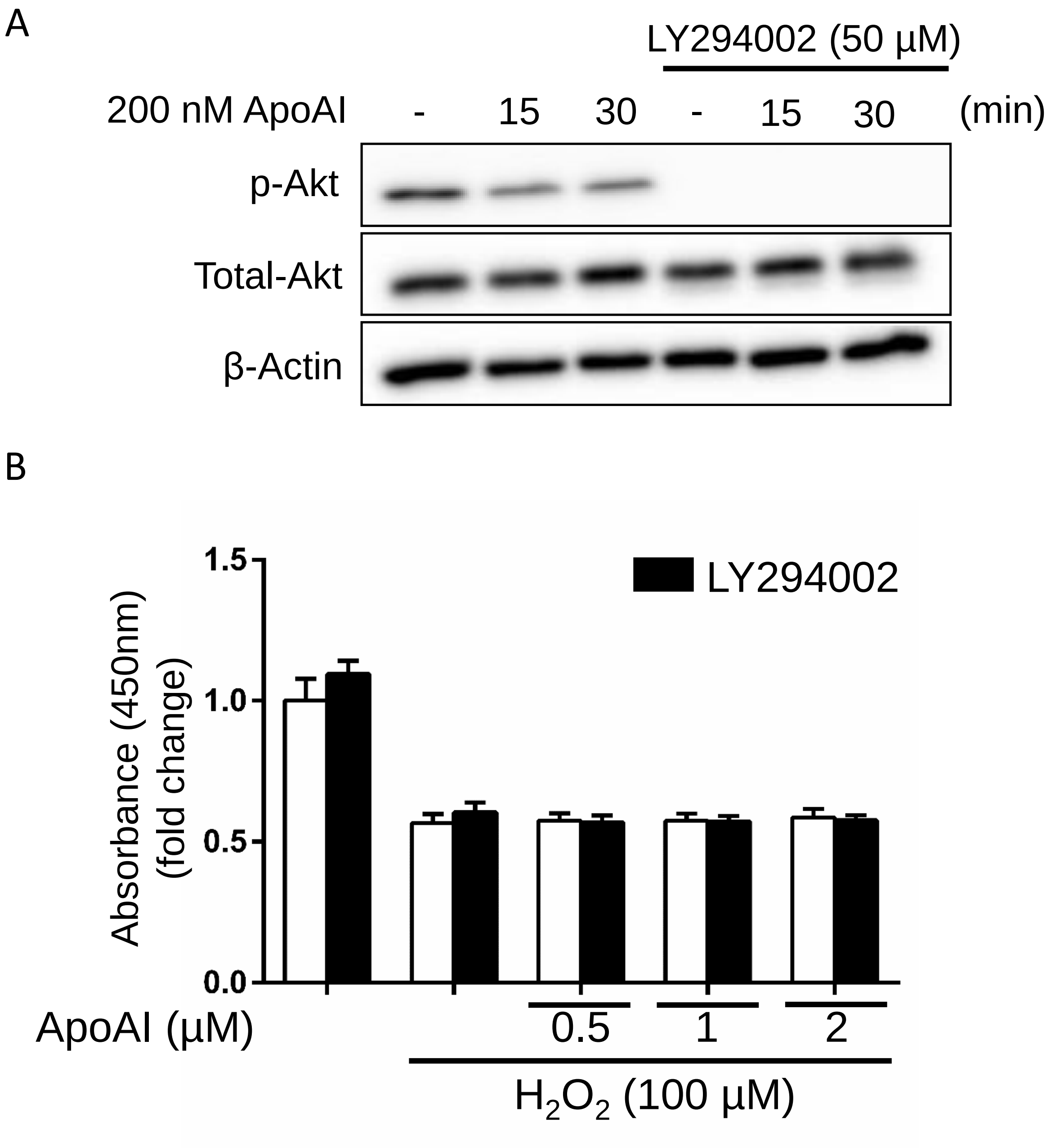

Supplemental Figure 3. **ApoAI does not affect cell survival in cardiomyocytes**

A, Western blot analysis of total and phospho-Akt in H9c2 cells. β-Actin serves as a loading control. Cells were incubated with 200 nM human ApoAI (SIGMA, USA) for 15 or 30 min with or without 50 µM LY294002.

B, After incubation with 0.5, 1, or 2 µM ApoAI overnight, cells were stimulated with 100 µM H_2O_2 for 2 hours with or without 50 µM LY294002. Cell viability was measured using a cell counting kit. (n=5 in each group). *, $P < 0.05$ by one-way ANOVA followed by Tukey's multiple comparison test. All values are mean ± SD.
